# 3D Printing Technology as a Promising Tool to Design Nanomedicine-Based Solid Dosage Forms: Contemporary Research and Future Scope

**DOI:** 10.3390/pharmaceutics15051448

**Published:** 2023-05-10

**Authors:** Javed Ahmad, Anuj Garg, Gulam Mustafa, Abdul Aleem Mohammed, Mohammad Zaki Ahmad

**Affiliations:** 1 Department of Pharmaceutics, College of Pharmacy, Najran University, Najran 11001, Saudi Arabia; 2 Institute of Pharmaceutical Research, GLA University, Mathura 281406, India; 3 Department of Pharmaceutical Sciences, College of Pharmacy, Al-Dawadmi Campus, Shaqra University, Shaqra 11961, Saudi Arabia

**Keywords:** 3D printing, pressure-assisted microsyringe (PAM), fused deposition modeling (FDM), solid dosage forms, nanomedicine, personalized medicine, SNEDDS

## Abstract

3D printing technology in medicine is gaining great attention from researchers since the FDA approved the first 3D-printed tablet (Spritam^®^) on the market. This technique permits the fabrication of various types of dosage forms with different geometries and designs. Its feasibility in the design of different types of pharmaceutical dosage forms is very promising for making quick prototypes because it is flexible and does not require expensive equipment or molds. However, the development of multi-functional drug delivery systems, specifically as solid dosage forms loaded with nanopharmaceuticals, has received attention in recent years, although it is challenging for formulators to convert them into a successful solid dosage form. The combination of nanotechnology with the 3D printing technique in the field of medicine has provided a platform to overcome the challenges associated with the fabrication of nanomedicine-based solid dosage forms. Therefore, the major focus of the present manuscript is to review the recent research developments that involved the formulation design of nanomedicine-based solid dosage forms utilizing 3D printing technology. Utilization of 3D printing techniques in the field of nanopharmaceuticals achieved the successful transformation of liquid polymeric nanocapsules and liquid self-nanoemulsifying drug delivery systems (SNEDDS) to solid dosage forms such as tablets and suppositories easily with customized doses as per the needs of the individual patient (personalized medicine). Furthermore, the present review also highlights the utility of extrusion-based 3D printing techniques (Pressure-Assisted Microsyringe—PAM; Fused Deposition Modeling—FDM) to produce tablets and suppositories containing polymeric nanocapsule systems and SNEDDS for oral and rectal administration. The manuscript critically analyzes contemporary research related to the impact of various process parameters on the performance of 3D-printed solid dosage forms.

## 1. Introduction

The solid dosage form is the most commonly prescribed and preferred method of drug administration, representing a significant portion of all prescriptions. This is because of high patient compliance, stability, ease of administration, handling, and transportation, high productivity, and low cost [1,2]. It is commonly administered through the oral route, which is the most preferred route of drug administration due to the possibility of self-administration, non-invasiveness, and better drug absorption to achieve local and systemic effects. A few of the solid dosage forms containing different pharmaceuticals are also administered through the rectal and vaginal routes. It avoids first-pass metabolism and gastrointestinal-related side effects to achieve local and systemic delivery of pharmaceutical products [3]. Commonly used solid dosage forms such as tablets, despite high productivity and convenient manufacturing coupled with technological advancement, are still based on conventional automated tablet presses, which rely on the same principle of tablet production with designs using dies and punches that have remained unchanged for decades [4]. Conventional tablet machines are still associated with limitations in producing flexible designs and tablet sizes to fulfill the current demand for personalized drug delivery approaches to produce customized doses with modulated drug release characteristics according to individual patients’ needs, which is the scope for future medicine in the near future [5,6,7]. The introduction of 3D (three-dimensional) printing technology has revolutionized the conventional drug manufacturing technique with its high flexibility in design according to size and on-demand production of tailored doses for customized drug delivery [8,9]. The discovery of a 3D printed product, Spritam (levetiracetam), in zip-dose technology by Aprecia Pharmaceuticals, approved by the US-FDA (United States Food and Drug Administration) in 2015, brought the focus of many researchers toward the use of 3D printing techniques as a novel and promising tool for the development of pharmaceutical products [10]. The 3D printing technique, also known as additive manufacturing, is a method of manufacturing a 3D object layer by layer using CAD (computer-aided design) [11,12]. The 3D printing technique results in the development of drug products with varied geometries and modified drug release characteristics, focusing on customized delivery of drug products [13,14]. The various types of 3D printing techniques that have been extensively used in the pharmaceutical field include deposition-based inkjet printing [15,16], powder bed deposition [17,18], extrusion-based fused deposition modeling (FDM) [19,20], pressure-assisted microsyringe (PAM) technique [21,22], laser-based selective laser sintering (SLS) [23], and stereolithography (SLA) techniques [24]. The application of nanotechnology in drug delivery has the potential to improve the biopharmaceutical attributes (improving the solubility and permeability) of drugs, enhance therapeutic efficacy, and reduce the side effects of APIs (active pharmaceutical ingredients). The 3D printing technique has been employed in the development of solid dosage forms, which exploit nanotechnology to fabricate nanomedicine-based solid dosage forms. The nanomedicine formulated through nanotechnology is further transformed into a solid dosage form by exploiting 3D printing technology as a nanomedicine-based solid dosage form. Extrusion-based 3D printing methods such as FDM and PAM are the most commonly employed techniques for the development of nanomedicine-based 3D-printed solid dosage forms. FDM is a fused deposition modeling method where the printing of thermoplastic material (in the form of filament) is carried out through an extruder nozzle of a 3D printer at an elevated temperature, which prints 3D objects/pharmaceutical products by holt-melt extrusion (HME) [25]. Whereas PAM is a pressure-assisted microsyringe technique where the printing material of semisolid consistency (such as pastes, gels, and wax) is filled in a syringe extruder to print a 3D object under pressure [26]. It is also known as the semi-solid extrusion (SSE) technique. SSE is basically a material extrusion 3D printing technology that can shape/print semi-solid materials into 3D objects/pharmaceutical products (such as tablets, suppositories, etc.) by extruding them through a heated orifice. Various nanocarrier systems, such as polymeric particles/nanoparticles [27], liposomes [28], lipid-based nanoemulsions [29], nanosuspensions [30], and SNEDDS [31,32], have been fabricated as 3D-printed pharmaceutical/biomedical products. The nanocarrier-based formulations were conventionally transferred as liquid injectables, filled-in oral capsules, or transdermal delivery systems such as nanogels, patches, etc. intended for patient administration. The emergence of 3D printing technology has provided a vital tool to develop these nanocarrier-based systems into solid dosage forms, especially in the form of oral 3D printed tablets [31,32] and also as suppositories [33] for local and systemic delivery of poorly soluble drugs. The 3D printing technology has successfully designed these nanocarrier-mediated drug delivery systems into a robust solid dosage form without hampering the nanonization characteristics of the loaded pharmaceuticals or therapeutics. The present manuscript is intended to provide a detailed discussion of the recent research developments that involved the formulation design of nanomedicine-based solid dosage forms utilizing 3D printing technology.

## 2. Rationale/Significance of 3D Printing Technology to Design Solid Dosage Forms

The development of nanomedicine-based formulations into a solid oral dosage form utilizing the 3D printing technique overcomes the limitations associated with conventional manufacturing methods. The 3D printing process requires a single processing step once the formulation feed (in the form of filament, ink, or paste) is ready, compared to the conventional tableting process [34]. The conventional manufacturing of solid dosage forms follows a sequence of steps such as mixing, granulation, drying, and compression. These processing steps may result in a change in nano characteristics, such as the size and shape of the nanocarrier system under processing. For example, polymeric nanocapsules that are conventionally formulated in suspension form are associated with physical stability problems due to microbial contamination [35]. However, developing a polymeric nanocapsule carrier system into a solid dosage form by the conventional tableting process may result in hampered nano characteristics of the system due to the high compression force applied in the conventional method. This may cause a change in nanocarrier size and shape, altering the properties of the system and its ability to deliver the drug in the form of a nanocarrier, thus influencing the efficacy of the system. Some efforts have been made to develop polymeric nanocapsules into solid form by freeze-drying [36], spray drying [35], and wet granulation [37]. However, there is one instance of developing a polymeric nanocapsule tablet by a conventional method of wet granulation that was reported to have low drug loading (0.083% *w*/*w*) [38]. Still, there is no report on developing freeze-dried or spray-dried polymeric nanocapsules into tablet form. Thus, converting polymeric nanocapsules into solid dosage forms is still a challenge for formulation scientists using conventional methods. Nevertheless, the 3D printing technique utilizing the hot-melt extrusion (HME) method successfully transforms a polymeric nanocapsule into a 3D printed solid dosage form [39]. In this study, the 3D-printed delivery device in the form of a tablet was soaked in the dispersion system of drug-loaded polymeric nanocapsules. It resulted in the fabrication of a 3D-printed tablet loaded with drug-containing polymeric nanocapsules. Similarly, the development of nanocarrier-based liquid SNEDDS into solid dosage form is conventionally by filling soft gel capsules or by adding an adsorbent to the liquid SNEDDS system and converting it to pellets or granules by spray drying or spheronization. However, filling soft gel capsules shows compatibility and stability issues such as leakage of the liquid SNEDDS from the capsule shell, change in taste perception, or softening or brittleness of the capsule shell [40]. However, the addition of an adsorbent to the liquid SNEDDS is also associated with a few drawbacks, such as dose dilution, hindered flow, and incomplete drug release from the adsorbed liquid SNEDDS system [41,42]. Moreover, efforts have been made to develop these liquid SNEDDS into tablet dosage form by direct compression, which may have resulted in a change in the size and shape of the carrier system due to the high compression force applied in the formulation development. In contrast, the 3D printing technique provides a rationale for the development of liquid SNEDDS into a 3D printed tablet dosage form by using FDM-based and PAM-based 3D printing techniques [43,44].

Nanomedicine-based formulations are intended to provide enhanced solubility, better stability, therapeutic efficacy, and improved bioavailability of APIs. Nanomedicine-based systems can be delivered in a specific dose according to the patient’s needs as personalized medicine. Specifically, a drug with a narrow therapeutic window that requires therapeutic drug monitoring is intended to be dose-customized. However, this dose customization has not been achieved by conventional techniques. For example, a SMEDDS/SNEDDS-based marketed product, Neoral^®^, was developed in a soft gel capsule containing cyclosporine (an immunosuppressant drug), available as a single dose in the form of “one-size-fits-for-all”. This immunosuppressant drug shows inter-subject variability in patients with organ transplants [45]. This requires personalization of the dose according to the patient’s needs. The 3D printing technique provides a solution to dose customization; based on an individual’s disease condition, differences in the genome, and metabolic pathways, customization of dose can be achieved [46]. Moreover, with the 3D printing technique, different geometries (size and shape) for the solid dosage forms can be designed, and tuned drug release characteristics could be achieved. The geometry of the dosage form influences the patient’s visual preferences in selecting a dosage form and thus enhances patient compliance [47]. With respect to this, a research group carried out a study in a clinical setting by printing a pediatric chewable formulation for a rare metabolic syndrome. The chewable formulations were printed in different flavors and colors, which were well accepted by the pediatric population, thus providing a way for dose customization at the point of care according to the patient’s needs [48].

## 3. 3D Printing Technology to Design Nanomedicine-Based Solid Dosage Forms: Contemporary Research

3D printing is a production technique in which a product is constructed from a digital model by depositing material layer-by-layer to form the final product. There has been a rise in curiosity about the use of 3D printing in the pharmaceutical industry ever since the FDA approved Spritam^®^ [49,50]. In the pharmaceutical industry, 3D printing has been used for drug discovery and the manufacture of drug delivery devices, while in biomanufacturing, it has been used for bone and tissue engineering via scaffolds [50,51]. This technique is also employed to fabricate 4D items from the programmed change of smart materials in response to environmental stimuli. Programmable materials are those that may change geometry and function in response to external stimuli such as heat, pH, light, or a magnetic field [52]. The 3D printing technique is a cutting-edge approach for producing products with a wide range of possible configurations [53]. This printing approach is accomplished by several different processes, including SLS, SLA, FDM, SSE, and powder bed-inkjet printing [54,55,56,57]. Okafor-Muo et al. summarize how 3D printing can be used to make pills for swallowing [58]. Although many studies focused on FDM because of its accessibility and versatility, other 3D printing technologies were discovered to be acceptable for the creation of oral solid dosage forms. The concept of building up an object from digital blueprints layer-by-layer is the basic concept of the 3D printing process. A diverse array of pharmaceutical excipients was successfully processed by the technique [59,60,61]. Smart materials utilized in the 4D printing technique for pharmaceutical product development can undergo programmable transformations within the formulated 3D objects [62,63,64]. Stimuli-responsive biomaterials and drug delivery systems are widely explored and have significantly influenced the drug pharmacokinetics and solubility characteristics of APIs [65,66].

**Figure 1:**
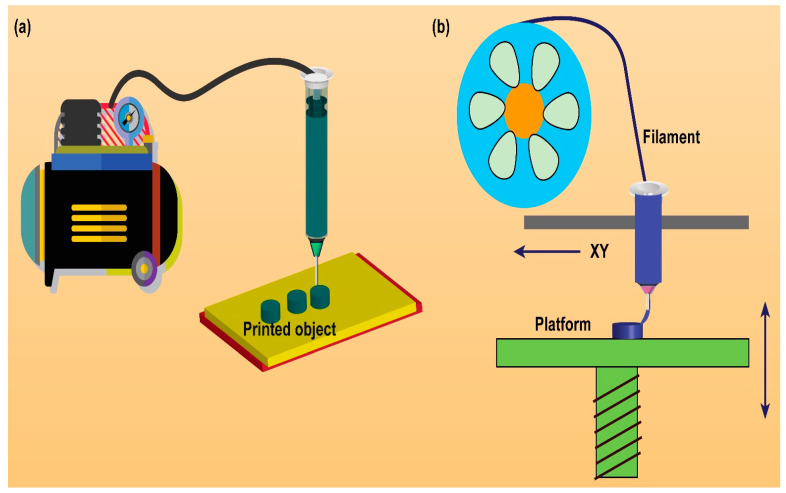
Schematic illustration of extrusion-based 3D printing technique utilized in the design and development of nanomedicine-based solid dosage forms. (**a**) Pressure-assisted microsyringe (PAM) or semisolid extrusion (SSE) technique. Pressurized air supply through a compressor to a movable piston, resulting extrusion of semisolid printing material through a printing nozzle to print a 3D object. (**b**) Fused deposition modeling (FDM) technique. Printing material in the form of filament is heated to a molten state and extruded through the printing nozzle to print a 3D object. Modified from Mohammed et al. [49], Elsevier, 2021.

The different types of 3D printing techniques are widely explored to design solid dosage forms with various intentions. Indeed, the extrusion-based (FDM, PAM) 3D printing technique was successfully implemented in the design of nanomedicine-based solid dosage forms (illustrated in Figure 1). The technique helps to retain the nano-dimension characteristics of nanomedicine after successful conversion into solid dosage forms.

Gioumouxouzis and co-workers tried to make 3D-printed dosage forms that could be controlled by osmosis using a water-permeable shell on the outside and an API in the osmotic core on the inside [53]. The formulation was designed using the FDM technique to build an exterior shell around an osmotically active core using cellulose acetate as the polymer. In FDM, a heated nozzle that moves along the X, Y, and Z axes lays down each layer of molten polymeric strands on the building plate. The shape of the dosage form was altered in response to the effect of the gradually generated osmotic pressure at various intervals of time. In formulation, amorphized diltiazem as a drug was combined with sodium chloride, which served as the osmogen. Diltiazem was chosen as a model drug for several reasons, the most important of which was its thermostability and the fact that it is a commonly prescribed prescription that is included in a wide variety of controlled-release formulations [67,68,69]. Three distinct formulations were produced, each with a different cellulose acetate shell shape. Specifically, one and two linear cavities were created for the shells of the first and second formulations, respectively, while the entire upper portion of the shell was removed from the third formulation. In addition, the upper and lower shell walls were perforated to promote water permeability. The aforementioned formulations demonstrated the feasibility of developing sophisticated in-situ drug delivery systems with patient-specific, tailored API (active pharmaceutical ingredient) content. In another investigation, McDonagh and his colleagues looked at how geometric scaling and pore structure affect the dose and release of drugs in 3D-printed solid dosage forms [70]. The study looks into how simple changes to software and design slicing can be used to meet both the dose and release requirements of the drugs. Paracetamol was used as a model drug to investigate the effects and interactions of the infill pattern and tablet geometry on the dose and release of the drug. An immediate-release erodible system and an immediate-release swellable system containing paracetamol were designed using Eudragit EPO and soluplus^®^ as polymeric excipients, respectively. Researchers studied the impacts of tablet height and diameter on drug release by printing constant-dose tablets with infill densities ranging from 30% to 90%. The rate of drug release from water-soluble and water-swellable polymers is determined primarily by the relative contributions of two mechanisms, i.e., diffusion and matrix dissolution or erosion. The surface area to volume (SA/V) ratio of the dosage form is known to have a significant effect on these mechanisms. The study revealed that the geometry scaling of eudragit EPO tablets with 30% to 70% infill density did not significantly influence the drug release rate of the tablets. With more than 90% infill density, it was found that geometric scaling had a significant effect on the drug release rate. Recently, the acceptability of 3D-printed solid dosage forms among patients was investigated in a clinical study. Bogdahn and co-workers investigated the geometry of 3D-printed solid dosage forms and the factors that influence their oral swallowability behavior [71]. In a controlled, randomized, crossover investigation, the swallowability of 3D-printed dosage forms of similar size and shape was evaluated. Twelve volunteers participated to assess six 3D-printed placebo objects and two compressed placebo objects (as references) in a double-blind study. Swallowing ability, feeling of a foreign body before swallowing, feeling of a foreign body after swallowing, and pain after swallowing were evaluated using a set of questionnaires. The oblong-shape tablets that were 3D printed and the compressed tablets are considered the best on all questions, followed by the round tablets that were also 3D printed. The pyramid-shaped tablets are considered the poorest in the ease of swallowability. This research demonstrated the importance of conducting swallowability studies on a wide range of 3D-printed solid dosage forms because not all of them are equally easy to swallow [71].

Personalized medicine entails tailoring drug treatment, dosing intervals, drug combinations, and drug release rates to account for individual needs [72]. Nanotechnology, on the other hand, has played a key role in the development of novel drug delivery systems to meet the needs of individuals, contrary to the ‘one-size-fits-all’ hypothesis. It has great potential to modify the biopharmaceutical attributes of poorly absorbable drugs. The development of multi-functional drug delivery systems, specifically solid dosage forms loaded with nanopharmaceuticals, has gained much attention utilizing 3D printing technology in recent years. Nanocapsules are physically stable for some months, but they pose a higher susceptibility to microbial contamination for long-term preservation due to their high water content [73]. Until recently, only a few efforts had been made to generate solid dosage forms, including nanocapsules, to solve limitations such as microbial spoilage, physical stability, storage, and transportation, using processes such as spray-drying, freeze-drying, and wet granulation. Similarly, liquid SNEDDS are converted to solid SNEDDS to improve their physical stability and patient compliance. In recent times, 3D printing technology has been widely explored to design nanomedicine-based solid dosage forms. The detailed perspectives of nanomedicine-based solid dosage form development utilizing 3D printing techniques are discussed in the subsequent section.

### 3.1. 3D Printed Tablets Loaded with Polymeric Nanocapsules

**Figure 2:**
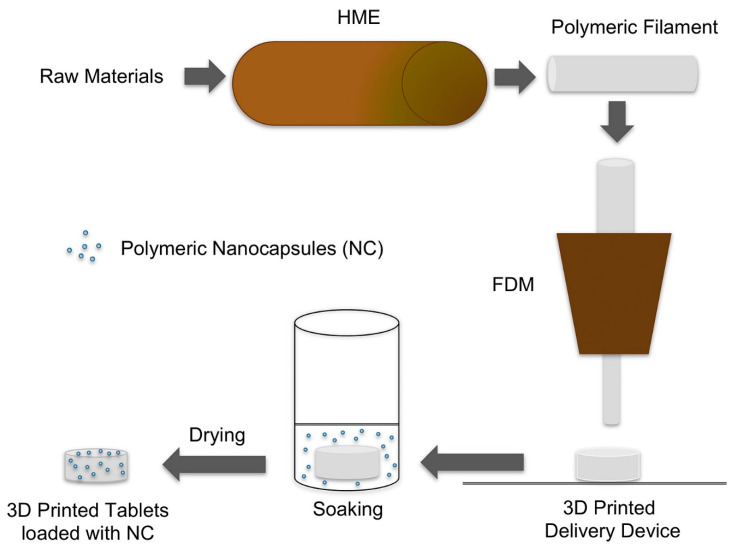
Schematic illustration highlights the design and development process to fabricate 3D printed tablet dosage forms loaded with polymeric nanocapsules. Reprinted with permission from Beck et al. [39], copyright 2017, Elsevier. HME—hot melt extrusion; FDM—fused deposition modeling; NC—nanocapsule.

The FDM-based 3D printing approach was utilized to transform the polymeric nanocapsule suspensions into tablet dosage forms to develop customized drug delivery systems for deflazacort [39]. A schematic illustration is shown in Figure 2.

Beck et al. combined 3D printing and nanotechnology for the first time in the production of tablet dosage forms containing polymeric nanocapsules as personalized medicines, allowing them to modify their drug dose and drug release profile [39]. 3D-printed tablets (printlets) containing nanocapsules were created using FDM from poly (e-caprolactone) and Eudragit1 RL100 filaments with or without a channeling agent (mannitol). The printed devices were drug-loaded by soaking them in a nanoparticle liquid suspension. Deflazacort was used as a model drug in the study. The presence of the channeling agent increased drug loading, and a linear association was found between soaking time and drug loading (r^2^ = 0.9739). The drug loading ought to be proportional to the volume of deflazacort nanocapsule suspension absorbed by the 3D-printed device during the soaking procedure. In this regard, the expected drug loading values were computed by taking into account the drug content of the deflazacort, the volume absorbed, and the final mass of the tablet. Furthermore, drug release profiles were affected by the polymeric nature of the tablets as well as the presence of the channeling agent. This study represented a revolutionary method for converting nanocapsule fluids into solid dosage forms as well as an efficient 3D printing technology for designing novel drug delivery systems for nanomedicine-based personalized medicine [39].

**Figure 3:**
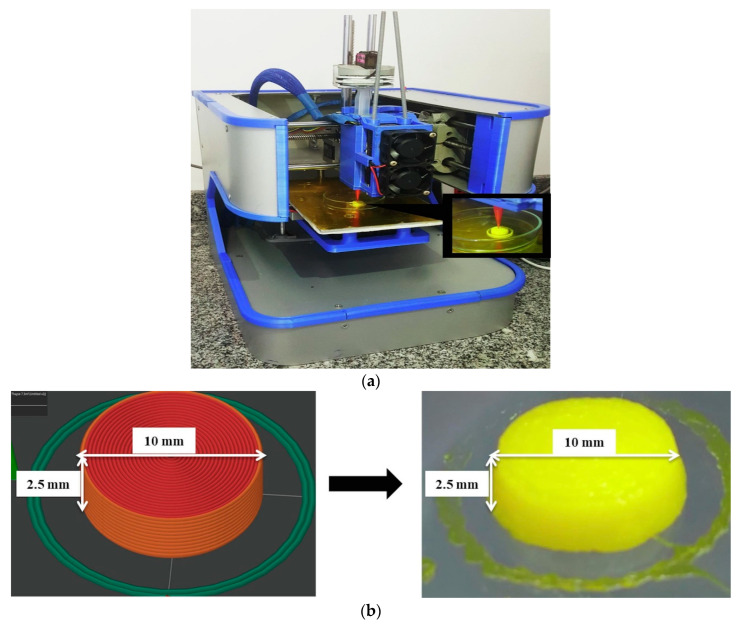
Schematic illustration represents (**a**) 3D printing of solid dosage form nanomedicine by SSE technique containing curcumin/resveratrol-loaded polymeric nanocapsules for oral administration. (**b**) Image view of G-CODE of the designed form (**left**) and 3D printed solid dosage form nanomedicine (**right**) before drying. Reprinted with permission from de Oliveira et al. [73], copyright 2022, Elsevier.

In another investigation, de Oliveira et al. fabricated a nanomedicine-based solid dosage form that consists of curcumin and resveratrol co-encapsulated in polymeric nanocapsules [73]. This 3D-printed nanomedicine-based solid dosage form was prepared by semisolid extrusion (SSE or PAM) technique (illustrated in Figure 3) with a disintegration time of <45 min and recovery of nanosized drug-loaded nanocapsules (particle size distribution in the range of 100–1000 nm) in a drug release study. This investigation provided a proof-of-concept related to the utilization of the SSE/PAM technique to fabricate solid nanomedicine for oral administration from a liquid suspension of nanocapsules.

### 3.2. 3D Printing of Self-Nanoemulsifying Tablets

**Figure 4:**
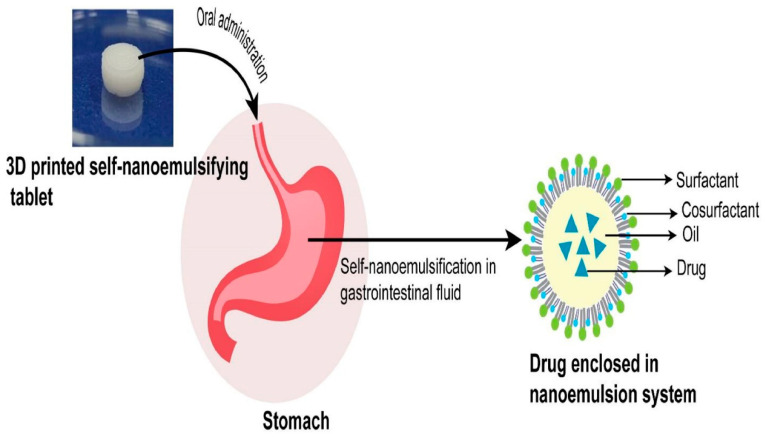
Schematic illustration represents the formation of a drug-encapsulated nanoemulsion system after self-nanoemulsification of a 3D printed tablet (nanomedicine-based solid dosage forms) in the stomach. Reproduced from Algahtani et al. [74], MDPI, 2021.

The concept of designing a lipid-based formulation technique, particularly a self-nanoemulsifying system for poorly water-soluble compounds, has received a lot of attention in recent years. It aids drug solubilization and improves the oral bioavailability of encapsulated drugs. It forms a stable nanoemulsion system after oral administration in the stomach (illustrated in Figure 4).

After the SNEDDS are dispersed in the aqueous medium, they self-emulsify and encapsulate poorly soluble drugs in oil droplets at the nanoscale. This makes the drugs easier to dissolve and absorb. Most of the SNEDDS products on the market were made as liquids that were put into soft gel capsules so they could be taken by mouth. Despite their benefits, liquid-based nanoemulsion formulations have several drawbacks, including limited stability. The widespread commercial development of liquid SNEDDS has been hampered as a result of these limitations, which has increased the need to transform these compositions into solid, self-nanoemulsifying dosage forms. A solid carrier (colloidal silica, dextran, microcrystalline cellulose, lactose, etc.) was used in the conventional solidification methods, which then utilized spray drying, spray cooling, and extrusion-spheronization to transform the SNEDDS solution into solid pellets [75,76]. To convert liquid SNEDDS into solid SNEDDS, these methods demand the inclusion of a sizable quantity of solid carriers. The self-nanoemulsification of SNEDDS is inhibited by the incorporation of higher quantities of solid adsorbent, and a huge proportion of surfactant may be required to facilitate self-nanoemulsification and the formation of colloidal dispersion upon contact with the aqueous phase [77,78]. The addition of huge volumes of adsorbent may potentially impact dosage uniformity and repeatability and cause safety concerns [79,80]. Additionally, some of the main problems in converting designed liquid SNEDDS to simple-to-handle solid SNEDDS include drug precipitation and phase separation. Several other issues need to be resolved, including poor transportability and poor palatability caused by high lipid contents [81]. SNEDDS face the difficulty of dosage dumping in the event of a poorly constructed system because they are fundamentally linked to a burst release [82]. This very successful technique is a poor choice for the sustained or controlled release needed to treat GIT (gastrointestinal tract) difficulties since it is challenging to distribute SNEDDS while controlling their release [83]. In the last few years, different researchers have used 3D printing techniques to turn these liquid SNEDDS into solid dosage forms.

Recently, PAM-based 3D printing was used to create a self-nanoemulsifying tablet of dapagliflozin propanediol monohydrate [74]. There are two primary components, viz., liquid and solid phases, to the formulation system that has been developed. The liquid phase of the developed formulation system is made up of oils (caproyl 90 and octanoic acid), a co-surfactant (PEG 400), and a solid matrix (PEG 6000). When the liquid phase comes into contact with the aqueous phase/gastrointestinal fluid, it self-nanoemulsifies into a drug-encapsulated nanoemulsion system. The solid phase was carefully selected based on its ability to self-nanoemulsify the preselected liquid phase system and its ability to fit the liquid phase system into its crystal lattice. As a surfactant and solidifying matrix for the preselected liquid phase, a mixture of poloxamer 188 and PEG 6000 in a 1:1 ratio was chosen. It was self-dispersible and could turn the chosen liquid system into a nanoemulsion when it came into contact with the aqueous phase. Due to its surface-active properties, Poloxamer 188 has also been used as a solubility-enhancing solid dispersion carrier for poorly water-soluble drugs that self-nanoemulsify as a drug-encapsulated nanoemulsion system when exposed to the aqueous phase/gastrointestinal fluid [84]. An in vitro drug release study revealed the faster release of poorly soluble drugs from developed 3D-printed self-nanoemulsifying tablets of various sizes (8 mm, 10 mm, and 12 mm). Smaller tablets (8 mm) released approximately 95% of the drug, while larger ones (10 mm or more) released 89% of the drug. The faster release from the smaller tablets than the larger ones could be attributed to their high SA/V ratio. Tablets with a high SA/V display a faster release rate due to the high level of interaction between the tablet’s surface and the surrounding medium. Hence, the study concluded that poloxamer 188 and PEG 6000, as emulsifying and solidifying agents, did not inhibit or delay the rate of self-nanoemulsification of solid-SNEDDS as the drug-lipid phase remained equally disseminated in the microstructure of the solidifying agents. Thus, 3D printing could be a promising potential technique to produce self-nanoemulsifying solid dosage forms for poorly water-soluble drugs without affecting their emulsification or biopharmaceutical attributes.

Vithani et al. investigated the use of 3D printing to create solid, self-microemulsifying drug delivery systems (S-SMEDDS) with a defined SA/V ratio and its effect on drug dissolution rate [44]. Due to their widespread use as lipophilic substances in a variety of investigations with lipid-based formulations and their poor water solubilities, fenofibrate (FEN) and cinnarizine (CINN) were chosen as model drugs. The study also wanted to show that 3DP technology can be used to make drug-loaded S-SMEDDS formulations without the need for a solid-phase carrier and allow for precise control of the release and absorption. Gelucire^®^ 44/14 (GEL 44 as a core digestible lipid), Gelucire^®^ 48/16 (GEL 48 as a surfactant), and Kolliphor^®^ P 188 (P 188 as a cosurfactant) were used as a core digestible lipid, a surfactant, and a cosurfactant, respectively, to create a variety of drug-loaded S-SMEDDS formulations with distinct geometries. The cylinder, prism, cube, and torus were chosen because of their varying SA/V ratios.

The results of the research unequivocally demonstrated the importance of geometry in dictating the dispersion process. Dispersion times for both drug-loaded systems were quickest for the torus, then the prism, the control cube, the cylinder, and finally the cube. Dispersion times were shown to be shorter for torus shapes (42 min and 58 min for FEN- and CINN-loaded S-SMEDDS, respectively) and longer for cube shapes (66 min and 81 min for FEN- and CINN-loaded S-SMEDDS, respectively) in general when SA/V ratio values changed. Hence, the study indicated that the dispersion kinetics of drug-loaded S-SMEDDS formulations depend on the SA/V ratio values. Additionally, the study showed that the drugs were not in an amorphous form in the final mixtures and that they were probably dissolved in the molten lipid-surfactant matrix at high temperatures and then crystallized again when the temperature dropped.

In their recent work, Kulkarni and co-workers showed how combining the two drug delivery platforms—3D printing and SNEDDS—would be helpful to develop new techniques for producing poorly soluble drugs while overcoming the previously stated challenges [85]. It would also open up new possibilities for personalized drug delivery with additional therapeutic advantages. By combining this platform with FDM 3D printing, novel approaches for delivering poorly soluble drugs and customized drug-delivery systems with enhanced therapeutic advantages could be achieved. The study detailed how pre-formulated SNEDDS of two drugs with low intrinsic solubility—curcumin (CUR) and lansoprazole (LNS)—were integrated into a hollow tablet utilizing an FDM-based 3D printing method [85]. Using the HME technique, a mixture of HPC and Soluplus in a 1:1 ratio created high-quality 3D-printable filaments with optimal physical and chemical qualities. These filaments are then utilized to prepare hollow tablets, which are then loaded with SNEDDs. The study confirmed that these SNEDDS were packed into the 3D-printed container without any degradation or leakage of the contents. Additionally, the new SNEDDS formula made it possible to delay the release of the drug from the hollow prints and change the thickness of the walls of the prints to produce lag phases. According to the study, future research into the filament used to prepare the container system could result in a more adaptable method of delivering any additional drugs. This helps with multi-drug regimens and incorporates many potential therapeutic classes. A blank filament consisting of a substance designed to release the drug at the location of drug absorption is possible. Drugs that are prone to breakdown at an acidic pH can be delivered more effectively with the use of pH-dependent release polymers. Moreover, depending on the needs of the therapy, a drug may be added to the filament. This study envisaged that combining these two unique drug-delivery techniques would solve multi-drug delivery and gastric pH degradation issues for treating illness conditions across the gastrointestinal tract (GIT). These delivery methods may also be effective for delivering numerous difficult compounds, such as biologics, to the target location, establishing a new framework for multi-drug delivery systems [85].

**Figure 5:**
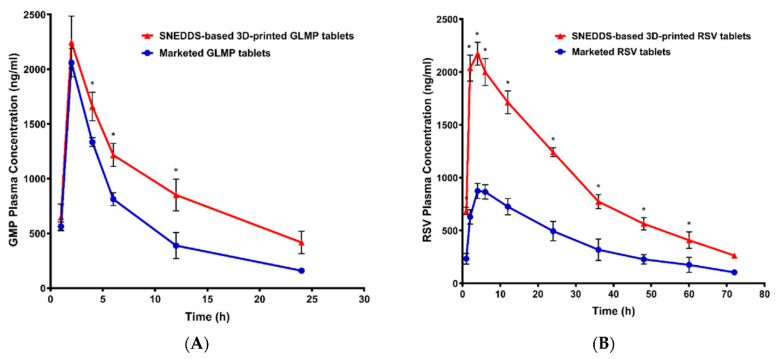
The graph illustrates plasma concentration versus time plot of 3D-printed SNEDDS-based tablet compared to marketed tablet after oral administration in Wistar rats. (**A**) Glimepiride. (**B**) Rosuvastatin. Reproduced from Ahmed et al. [86], MDPI, 2021. SNEDDS—Self-nanoemulsifying drug delivery system; GLMP—Glimepiride; RSV—Rosuvastatin. * Indicates significant difference (at *p* < 0.05) between groups.

In another investigation, the fabrication of 3DP multi-compartment tablets incorporating glimepiride (GLMP) and/or rosuvastatin (RSV) loaded-SNEDDS formulations was studied by Ahmed et al. [86]. Different SNEDDS formulations using Tween 80 and PEG 400 as the surfactant and cosurfactant, respectively, were prepared using the oil of Curcuma longa. HPMC (4% *w*/*v*) was utilized as a gelling agent in the preparation of both SNEDDS-based and non-SNEDDS gel formulations. Furthermore, to create the adsorbent for the SNEDDS formulation, PVPK90, lactose, and methocel were combined and placed in a mortar with the gel matrix. Additionally, Ac-Di-Sol and Avicel were used as disintegrants and insoluble ingredients, respectively. The ingredients were combined until a smooth, uniform paste was produced. Pseudoplastic shear-thinning flow behavior was observed in the produced pastes. Extrusion printing was utilized to create 3DP tablets from the produced pastes. The successful printing of flat-faced round tablets with a 15 mm diameter and a 5.6–11.2 mm thickness demonstrated good characteristics for friability, weight fluctuation, and content homogeneity. The 3D printed tablets were examined using scanning electron microscopy, which indicated that the interior portion of the tablets had a gel-like porous structure and a semi-porous surface that showed some curvature and the appearance of tortuosity. When compared to tablets without SNEDDS, the release of the drug from SNEDDS-based tablets was superior. GLMP and RSV demonstrated relative bioavailability of 159.50% and 245.16%, respectively. The pharmacokinetic properties of the prepared 3D printed tablets were investigated in Wistar rats and compared with marketed tablets (as shown in Figure 5). The results showed that the developed 3D-printed tablets were superior in terms of absorption rate and extent. When GLMP and RSV were compared to the commercially available drugs, the 3DP tablets demonstrated higher maximum plasma concentrations across time. The relative bioavailability of GLMP and RSV was 159.50% and 245.16%, respectively. This result is explained by the fact that each drug is now available in a nanoform, which improves drug absorption from the gastrointestinal tract.

Later on, the same research group evaluated the pharmacokinetic and pharmacodynamic performance (including lowering of blood glucose level and lipid profile) of SNEDDS-based 3D printed tablets in streptozotocin/poloxamer-induced diabetic/dyslipidemic rats [87]. The antidyslipidemic, hepatoprotective, and antioxidant actions of the developed 3D-printed tablets based on SNEDDS were also significantly higher than those of the placebo and non-SNEDDS-loaded tablets. By increasing HDL and decreasing triglycerides and LDL, polypills containing SNEDDS for GMD/RSV may reduce the risk of cardiovascular disease and atherosclerosis. Hepatic sinusoids were normal, and there were no obvious abnormalities in histopathology, indicating the maximum level of protection possible. Microscopic analysis of the pancreatic material revealed islets of normal size as well as exocrine acini and the pancreatic duct, both of which were consistent with normal pancreatic parenchyma. These results corroborate the efficacy of curcuma oil in reducing the risk of cardiovascular disease and liver steatosis in patients with metabolic syndrome when used in combination with GMD and RSV. There has to be more clinical research done to back up the current findings. These 3D-printed tablets containing SNEDDS of GLMP and RSV improved the pharmacokinetics of both glimepiride and rosuvastatin, with AUC_0–t_ values of 16,035.25 ng/mL.h and 67,811.75 ng/mL.h, respectively, in streptozotocin/poloxamer-induced diabetic/dyslipidemic rats, similar to the previous study. Thus, these studies concluded that the developed 3DP tablets based on SNEDDS could be considered a promising combination oral drug therapy used in the treatment of metabolic disorders. However, clinical studies are needed to investigate their efficacy and safety [86,87].

Recently, 3D-printed tablets comprising *Nigella sativa* oil (black seed oil)-based SNEDDS of glimperamide have been formulated to overcome the poor aqueous solubility and limited dissolution of glimperamide in the GIT fluids and to avoid dose dumping [88]. The study reported black seed oil SNEDDS containing glimperamide for the first time. The study fabricated glimepiride tablets using three different manufacturing techniques, viz., direct compression, liquidsolid, and SNEDDS-based 3D printed tablets using the extrusion method, and subsequently compared their quality attributes and pharmacokinetic properties. The three-dimensional printing tablets had some porous surfaces, whereas the liquisolid and directly compressed tablets had smooth, non-porous surfaces. The inner structure of the three-dimensional printing tablets had some tortuosity and a gel-like porous structure, whereas the inner structure of the liquisolid tablets revealed some cracks and spaces between the included tablet materials. The liquisolid and 3D tablets improved most of the computed pharmacokinetic characteristics. While the three-dimensional tablets displayed regulated drug release behavior, the liquisolid tablets displayed rapid in vitro drug release. The tablets’ surfaces and internal structures showed noticeable variances. The majority of the computed pharmacokinetic parameters improved in the liquisolid and three-dimensional printed tablets. The liquid-solid and three-dimensional printed tablets are therefore potential methods for altering glimepiride release and enhancing in vivo performance, although additional clinical studies are needed [88].

### 3.3. 3D Printing of SNEDDS-Based Suppositories

The conventional preparation of suppositories involves a molding process that takes multiple phases and a considerable period of hardening. When there isn’t a suppository formulation, hospital pharmacists often make suppository formulations from pills that are meant to be taken by mouth. This results in a multitude of dangers, including incorrect compounding and dosing. Given these concerns, 3D printing has been investigated as a revolutionary manufacturing technology for the production of customized formulations, such as suppositories. With the implementation of 3D printers in hospitals, suppository sizes can be tailored to the patient’s comfort. Thus, suppository systems for personalized drug delivery could be conceived of as an innovative application for 3D printing. Moreover, given that discomfort is the leading cause of non-adherence to suppositories in the treatment of ulcerative colitis, the capacity to customize suppositories to the patient’s comfort can improve adherence and, subsequently, treatment outcomes. Additionally, improved mucosal drug administration methods have been invented using 3D printing, with a significant emphasis on dosage personalization and product design flexibility.

Millions of people all around the world are affected by ulcerative colitis, making it a major public health issue. Rectal administration of immunosuppressive medicines, such as tacrolimus, is a potential technique to optimize drug concentration at the site of action while minimizing systemic side effects, as Crohn’s disease is an inflammatory disorder localized in the large intestine. Drugs for ulcerative colitis are difficult to administer orally since only a small percentage of the dose may reach the colon, where it is needed. Instead, rectal administration has been shown to improve therapeutic efficacy and safety by increasing medication concentrations at the illness site, decreasing systemic adverse effects, and speeding up the rate of response.

The PAM-based 3D printing technology has just recently been investigated for the formulation of SNEDDS-based lidocaine suppositories for individualized medication delivery [89]. The model drug lidocaine is metabolized rapidly by the liver, and metabolites and unchanged drugs are excreted by the kidneys. Researchers used a pressure-assisted microsyringe (PAM) technique to create custom lipid-based suppositories that could contain varying concentrations of lidocaine-free bases. The research made use of the thixotropic properties of Geloil^TM^SC to create printable ink. Adopted lipid oil-containing surfactants (Geluicire 48 and Kolliphor RH 40) were used to incorporate lidocaine at concentrations of 2–4% *w*/*w* and then tested for their physicochemical properties and their resemblance to the SNEDDS. The research recommended using a 10:90 oil-to-surfactant ratio to produce small nano-emulsion-size particles and reduce oil loss during storage. Further, DSC (differential scanning calorimetry) and XRD (X-ray diffraction) analysis revealed the presence of lidocaine in the amorphous state in the prepared printable ink. The SNEDDS-like suppositories met all of the criteria set forth by the European Pharmacopoeia in terms of their mechanical qualities and in vitro performance. In vitro dissolution profiles demonstrated that 2% *w*/*w* lidocaine-loaded suppositories released LID more slowly than those containing 4% *w*/*w* lidocaine. The slow release from the 3D-printed suppository containing 2% *w*/*w* lidocaine could be attributed to the formation of hydrogen bonds due to the presence of a high proportion of surfactants, and hence intermolecular interactions lead to a dense network. The drug release from developed 3D-printed suppositories follows Hixon–Crowell release kinetics, indicating that the formulation was affected by erosion. The study demonstrated the viability of 3D printing as an alternative method for creating tailored lidocaine-loaded suppositories for successful pain reduction during prostate biopsy [89].

Using 3D printing technology, Seoane-Viano and co-workers demonstrated that it was possible to manufacture tacrolimus-loaded suppositories without molds or other physical support [90]. Coconut oil and one of two lipids (Gelucire 44/14 or Gelucire 48/16) were used to print the suppositories vertically in three different sizes using a pharmaceutical semi-solid extrusion 3D printer. The suppositories hardened during the printing process in less than a minute, so they didn’t need to be chilled or dried any further. Since its melting point could not be found, tacrolimus may form a solid solution with the lipid excipients in the formulations. Tacrolimus was delivered at a rate of more than 80% within 120 min by both suppository devices; however, the rates of release varied greatly between the two. Tacrolimus’ immune-suppressive effects can be maintained while the risk of side effects is kept to a minimum by administering precise amounts of the drug in the target area. With 3D printing’s extraordinary adaptability comes the possibility of new avenues for the mass customization of dosage formulations [90].

**Figure 6:**
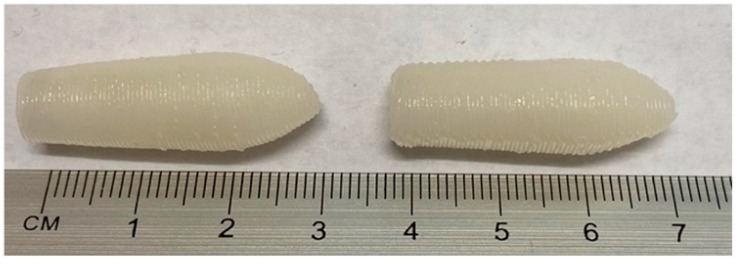
Infliximab-containing suppositories (GEL48W7.5 [**left**]—composed of Gelucire 48/16, coconut oil, water, and infliximab; GEL48W20 [**right**]—composed of Gelucire 48/16, water, and infliximab) prepared by SSE-based 3D printing techniques for rectal delivery. Reproduced from Awad et al. [91], copyright 2023, Elsevier.

**Figure 7:**
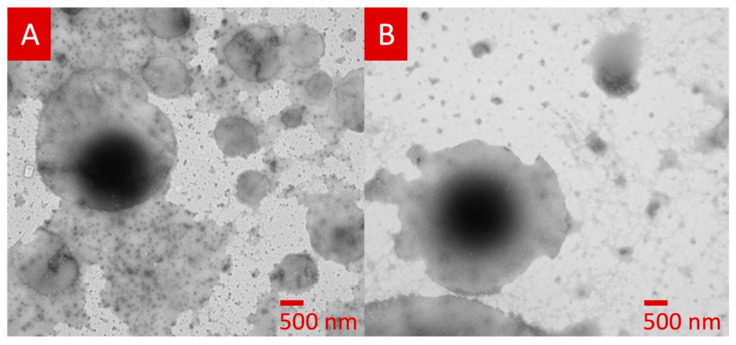
TEM image of lipid droplets formed following self-emulsification of GEL48W7.5 suppository (**A**) and GEL48W20 suppository (**B**). Reproduced from Awad et al. [91], copyright 2023, Elsevier.

Recently, Awad et al. fabricated infliximab (a monoclonal antibody) containing a suppository (shown in Figure 6) by 3D printing the SSE technique for rectal delivery for the treatment of inflammatory bowel disease [91]. It was observed that infliximab remains stable following the 3D printing process, and the GEL48W20 suppository disintegrated significantly faster (*p* < 0.05) than the GEL48W7.5 suppository. TEM images of lipid droplets formed following self-emulsification of formulated suppositories are spherical in shape (shown in Figure 7). It confirms the utility of 3D printing technology for the fabrication of solid dosage forms containing biopharmaceuticals.

**Figure 8:**
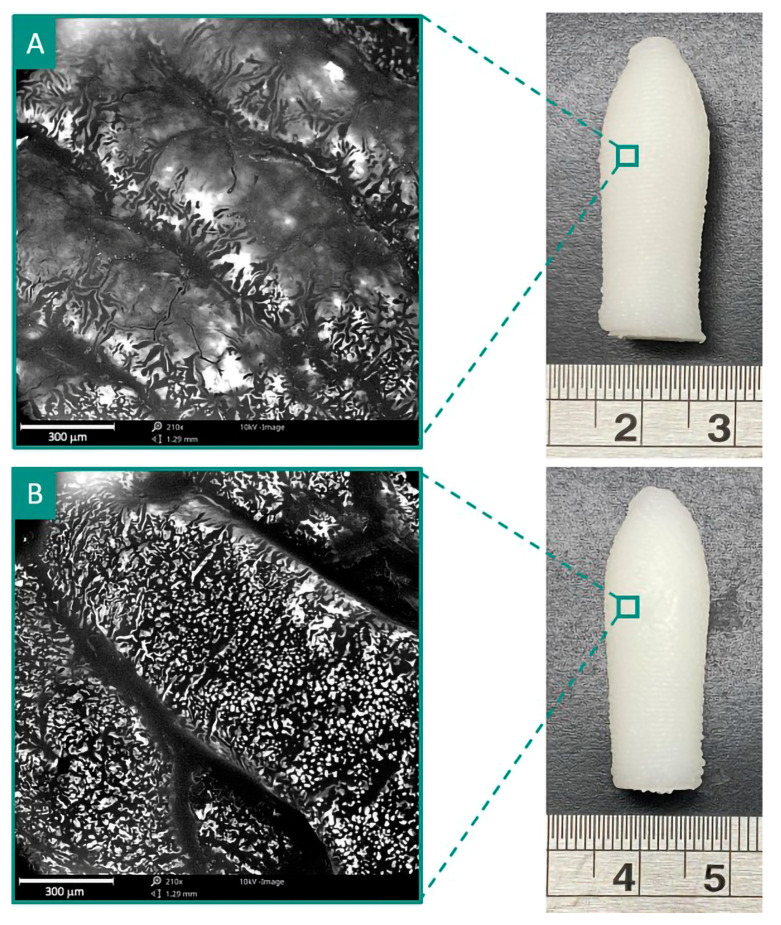
Scanning electron microscopy (SEM) image (**left**) and photograph (**right**) of 3D printed multi-drug-loaded suppository by SSE technique reveals good structural integrity between layers. (**A**) Suppository TOF10-BUD4, containing tofacitinib (10 mg) and budesonide (4 mg). (**B**) Suppository TOF5-BUD2, containing tofacitinib (5 mg) and budesonide (2 mg). Reproduced from Awad et al. [92], copyright 2023, Elsevier.

In another investigation, Awad et al. fabricated a multi-drug (budesonide and tofacitinib citrate as anti-inflammatory agents) loaded suppository (shown in Figure 8) by 3D printing the SSE technique for the treatment of acute severe ulcerative colitis [92]. The developed local delivery system is helpful in improving the therapeutic outcomes of inflammation localized in the rectum and colon. It permits the delivery of multiple drugs of a poorly soluble nature in personalized dosage forms.

The technique overview and process parameter-related summary of contemporary research carried out in the field of nanomedicine-based solid dosage forms utilizing 3D printing technology are shown in Table 1.

Furthermore, the outcome/purpose-related summary of contemporary research carried out in the field of nanomedicine-based solid dosage forms utilizing 3D printing technology is shown in Table 2.

## 4. Opportunity, Challenges, and Future Scope

Various trials for patient-friendly medications and medical devices have been developed by using computer-aided design software in the manufacturing of pharmaceuticals and medical equipment. This promoted the popularity of the new technique and offered a feasible substitute for the problems with conventional drug manufacturing. In the future, the 3D printing technique will be one of the precise methods used to provide tailored medicine, altering the dosage and administration profile to suit the needs of the patient [93]. To manufacture organs or an organ on a chip for drug testing, 3D printing can accurately manage the distribution of cells, extracellular matrix, and biomaterials. Complex structures that improve drug absorption or lessen negative drug interactions could be generated using computer-aided design [93]. However, several administrative and technological hurdles must be addressed to utilize this technology in the personal distribution of medications. In accordance with the World Health Organization (WHO), half of the medications prescribed are sold improperly, and half of the patients do not follow their prescribed treatment regimens. Wasted drugs have a detrimental effect on both the economy and the environment. The US precision medicine initiative was launched to understand how a patient’s genetics, environment, and lifestyle can help determine the best approach to prevent or treat disease [94]. Personalized medicine has also been placed at the forefront of the UK healthcare agenda. In 2016, the NHS published a report improving outcomes through personalized medicine, and more recently, the UK government published the policy paper ‘Genome UK: the future of healthcare, 2020′ [95] and the ‘Life Sciences Vision, 2021′ [96], showing a major affirmation in personalized medicine utilizing new technology to tailor the drug delivery approaches. 3D-printed medications have a huge future market, which is expected to increase from USD 638.6 million to USD 2064.8 million from 2019 to 2027, with a compound annual growth rate of 15.2% [97]. Hence, to have good reliability and clinical outcomes, regulating agencies need to put in place strict regulations. There are many challenges to be overcome to achieve widespread integration in the drug manufacturing industry, including regulatory standards, accumulating evidence to support safety and efficacy, and creating dosage forms and 3D printers that are suitable for use in the pharmaceutical industry as well as other stakeholders. A few technological challenges still need to be resolved before the technique can be extensively used. At present, there is a shortage of appropriate materials and excipients specifically developed for the production of 3D-printed medications. Similarly, 3D printing technology faces several notable limitations, including expensive pre- and post-processing costs, a restricted range of options, and technical and mathematical constraints. Furthermore, the use of 3D-based drug delivery in decentralized locations will have some legal and ethical issues, such as patient data protection and security, the risk of counterfeit production, and patient safety and liability considerations. Furthermore, there is a need for pharmacovigilance practices and policies to ensure adverse effects are appropriately identified and addressed. The application of 3D printing in the medical field is still in its infancy; however, the FDA has been the forerunner in its implementation and has issued technical guidance for the additive manufacturing of medical devices. The FDA has launched several internal management sciences and research improvements across the centers in response to increased good manufacturing practices, vigilance, and inquiry into 3D printed drug product goods, which are still in their developmental stage [98]. The quality assurance of 3D-printed dosage forms is a concerning area, and the FDA and pharmacopeias need to develop and disseminate quality standards that are better suited for ensuring the effectiveness of 3D-printed formulations.

## 5. Conclusions and Future Directions

The 3D printing technology could be successfully applied to transform the liquid polymeric nanocapsule system and liquid SNEDDS into solid dosage forms for oral (such as a tablet) and rectal (such as a suppository) administration. In this review, different research investigations provided evidence for the successful transformation of liquid SNEDDS into solid dosage forms without the use of solid adsorbents. Moreover, the utilization of 3D printing technology is providing a platform that could facilitate the easy and rapid fabrication of nanomedicine-based solid dosage forms. Other solid dosage forms are readily available in the literature (such as bio-active patches, mucoadhesive films, and modular systems) and should also be utilized to convert them into nanomedicine-based solid dosage forms exploiting 3D printing technology. Furthermore, the 3D-printed nanopharmaceutical products could be used for personalized medicines as well as to improve the bioavailability/biopharmaceutical attributes of poorly soluble drugs. The review also highlighted the significance of 3D printing process parameters on the performance and outcomes of the developed pharmaceutical products. However, there was still a lack of in-depth investigation into this interesting/promising area. The literature review indicated that clinical investigations are yet to be conducted for their successful applications in humans. As a result, in-depth investigations are still required in the area of 3D printed nanomedicine-based solid dosage forms to conduct detailed clinical investigations for their successful translation into clinical settings.

## Figures and Tables

**Table 1:** Contemporary research summary related to technique overview and process parameters involved in 3D printing-based nanomedicine containing solid dosage forms.

3D Technique	Process Parameters	Type of Solid Dosage Forms	Dimension and Other Characteristics of 3D Printed Dosage Forms	Ref.
FDM	- The extrusion nozzle die: 1.5 mm (ERL filaments) or 2.0 mm diameter (PCL filaments). - The extruding temperature 110 ± 5 °C for ERL filaments and 65 ± 5 °C for PCL filaments.	Tablet	- Number of shells = 2 - Infill percentage = 100% - Layer thickness = 0.20 mm	[73]
PAM	- Nozzle size: 0.84 mm - Printing speed: 10 - Printing pressure: 60 PSI	Tablet	3D Tab A: 8 mm × 3 mm 3D Tab B: 10 mm × 3 mm 3D Tab C: 12 mm × 3 mm	[74]
FDM	- Printing nozzle temp: 65 °C - Nozzle diameter: 1.5 mm - Nozzle speed: 3 mm/s - Layer height: 0.4 mm	Tablet	FEN S-SMEDDS: (L × W × H × Layers) - Cylinder: 11 × 11 × 3.3 × 9 - Prism: 12.3 × 12.3 × 3.8 × 11 - Cube: 7.3 × 7.3 × 5.5 × 15 CINN S-SMEDDS: (L × W × H × Layers) - Cylinder: 9.1 × 9.1 × 2.5 × 8 - Prism: 12 × 12 × 3.5 × 9 - Cube: 7 × 7 × 4.5 × 12	[85]
FDM	- Infill speed: 10 mm/s - Flow speed: 2.6 mm/s - Nozzle: 0.58 mm	Tablet	- Thickness: 5.797 ± 0.156 - Diameter: 14.342 ± 0.109 - Drug content: 4.052 ± 0.087 and 9.711 ± 0.097	[86]
FDM	- Infill speed: 10 mm/s - Flow speed: 2.6 mm/s. - Nozzle: 0.58 mm	Liqui-Solid tablet	- Thickness: 2.826± 0.228 - Diameter: 15.201 ± 0.228 - Drug content: 9.76 ± 0.359	[87]
FDM	- Flow speed: 2.6 mm/s - Nozzle: 0.58 mm	Liqui-Solid tablet	- Thickness: 3.239 ± 0.178 - Diameter: 14.855 ± 0.319 - Drug content: 1.92 ± 0.219	[88]
PAM	- Flow speed: - Nozzle size: 1.27 mm	Suppository	- Dimension: 2 × 15 × 20 mm - Arc: 45° arc (radius: 8.366 mm)	[89]
PAM	- Flow speed: 25 mm/s - Nozzle size: 1.2 mm	Suppository	- Layer height: 0.5 mm - Thickness: 2.4 mm	[90]

**Table 2:** Contemporary research summary related to outcome/purpose of nanomedicine-based solid dosage forms utilizing 3D printing technology.

Types of Nanocarriers	Drugs	Route of Administration	Outcome/Purpose	Ref.
Nanocapsules	Deflazacort	Oral	Partially hollow core with higher drug loading and faster drug release rate.	[73]
SNEDDS	Dapagliflozin	Oral	PAM-based 3D printing technique was found effective to print a self-nanoemulsifying tablet dosage form with an immediate-release drug profile for poorly water-soluble drugs.	[74]
SMEDDS	Fenofibrate or cinnarizine	Oral	The kinetics of dispersion depended on the SA/V ratio values. The digestion process was affected by the initial geometry of the dosage form by virtue of the kinetics of dispersion of the dosage forms into the digestion medium	[85]
SNEEDS	Glimepirideand rosuvastatin	Oral	Enhancement in the pharmacokinetic behavior when compared to the commercial tablets	[86]
SNEEDS	Glimepiride (GMD) and rosuvastatin	Oral	3D printed polypills improved the pharmacokinetics of both glimepiride and rosuvastatin with AUC values of 16,035.25 ng/mL × h and 67,811.75 ng/mL × h respectively.	[87]
SNEEDS	Glimepiride	Oral	SNEDDS formulation in the prepared paste lubricates the solid particles and prevents water loss. Fast in vitro drug release and controlled drug release behavior. 3D tablets showed an improvement in pharmacokinetic parameters compared to the directly compressed tablets with higher relative bioavailability.	[88]
SNEDDS	Lidocaine	Rectal	Work highlighted the potential of 3D printing as an alternative pathway to formulate personalized LID-loaded suppositories for local anesthesia	[89]
SNEDDS	Tacrolimus	Rectal	Fabrication of 3D printed self-supporting suppositories to deliver personalized doses of a narrow therapeutic index drug, with potential benefits for patients with ulcerative colitis	[90]

## Data Availability

Not applicable.
